# Prenatal Interventions for Congenital Diaphragmatic Hernia: A Systematic Review

**DOI:** 10.7759/cureus.86502

**Published:** 2025-06-21

**Authors:** Abdulaziz S Altala, Mohayad Ahmed, Ahmed O AlGhamdi, Fares Alroudhan, Rakan A Alqahtani, Faisal Alanazi, Khalid A Alshahrani, Zain Amer Alruwili, Imtenan A Oberi, Alhanouf Alqahtani, Amal N. Alamri, Judy Binyamin, Manar Alzahrani, Abdullah F. Alzebali, Khaled H AlFaifi

**Affiliations:** 1 Department of Basic Medical Sciences, College of Medicine, Dar Al Uloom University, Riyadh, SAU; 2 Department of Anatomy, Nile University, Khartoum, SDN; 3 Faculty of Medicine, Prince Sattam Bin Abdulaziz University, AlKharj, SAU; 4 Faculty of Medicine, Dar Aluloom University, Riyadh, SAU; 5 Faculty of Medicine, King Khalid University, Abha, SAU; 6 Faculty of Medicine, Northern Border University, Arar, SAU; 7 Faculty of Medicine, Jazan University, Jazan, SAU; 8 Faculty of Medicine, Imam Mohammad Ibn Saud Islamic University, Riyadh, SAU; 9 Faculty of Medicine, Ibn Sina National College, Jeddah, SAU; 10 Faculty of Medicine, Al-Baha University, Al-Baha, SAU; 11 Faculty of Medicine, King Abdulaziz University, Riyadh, SAU; 12 Faculty of Medicine, King Saud Medical City, ​Saudi Commission for Health Specialties, Riyadh, SAU

**Keywords:** congenital cardiac defect, congenital diaphragmatic hernia (cdh), fetoscopic endoluminal tracheal occlusion, maternal and neonatal outcomes, mother outcomes, neonatal intensive care unit (nicu) admission, premature preterm labor rupture of membranes (pprom), prenatal intervention, pulmonary hypoplasia

## Abstract

Life-threatening congenital diaphragmatic hernia (CDH) is a defect with subsequent herniation of abdominal organs. Herniation of abdominal organs into the thoracic cavity causes pulmonary hypoplasia and hypertension. Despite improved baby critical care and surgical therapy, severe CDH has high mortality and morbidity rates. The prenatal intervention of fetoscopic endoluminal tracheal occlusion (FETO) has become popular for improving postnatal survival and lung development. However, its efficacy in reducing maternal risks, morbidity, and improving newborn survival is still debated. This systematic study compares FETO with expectant management of mothers on neonatal and maternal outcomes.

A PICO-based systematic review was conducted. The study included fetuses with severe or moderate CDH. The intervention group had FETO, while the control group had expectant management with postnatal surgery. Neonatal survival, morbidity, and maternal problems were evaluated. A complete PubMed, OpenAlex, and CENTRAL search yielded 669 records. Forty papers met Preferred Reporting Items for Systematic Reviews and Meta-Analyses (PRISMA) eligibility criteria after deleting 154 duplicates, screening 515 abstracts, and analyzing 102 full-text articles. RCTs, cohort studies, and retrospective analyses were conducted. The data were extracted after title and abstract screening, followed by full-text screening using inclusion and exclusion criteria. Statistical analyses were then performed using RStudio (Posit PBC, Boston, MA, USA) to assess morbidity patterns, maternal risk factors, and pooled survival rates.

Pooled analyses suggest that FETO may be associated with improved survival in severe CDH cases with liver herniation compared to expectant management. Some studies report noticeably higher survival rates with FETO, though others have shown the opposite trend, possibly reflecting differences in patient selection criteria. FETO has also been associated with higher incidences of pulmonary hypertension, prolonged ventilatory support, and increased risk of gastrointestinal complications such as feeding difficulties, gastroesophageal reflux, and reherniation.

The impact of FETO on extracorporeal membrane oxygenation requirements appears inconsistent across studies, with some indicating a reduced need and others reporting similar rates regardless of intervention. A notable concern with FETO is its association with increased risks of preterm premature rupture of membranes (PPROM) and preterm delivery. PPROM has been reported in nearly half of FETO cases, compared to lower rates in expectant management. Correspondingly, gestational age at delivery tends to be earlier in FETO pregnancies, potentially contributing to lower birth weights and higher rates of neonatal intensive care unit admission. Serious maternal complications, such as hemorrhage, sepsis, or organ injury, are infrequently reported.

FETO improves survival in severe CDH cases; however, this systematic analysis demonstrates that treatment increases neonatal morbidity and maternal morbidity. The findings highlight the importance of precise patient selection to maximize benefits and minimize risks. Clinically, FETO should be reserved for severe CDH cases in which the survival benefits outweigh the problems. Future research should focus on standardizing FETO procedures, enhancing postnatal care, and investigating other therapies to reduce PPROM and preterm birth risk.

## Introduction and background

Description of the condition

Congenital diaphragmatic hernia (CDH) is a developmental defect in which the diaphragm is deficient, allowing abdominal organs to herniate into the thoracic cavity while the person is still in the womb. This displacement throws off normal lung development, which finally causes pulmonary hypoplasia and hypertension. These two diseases are the leading causes of neonatal morbidity and death rates [1]. The degree of the pulmonary damage largely determines the clinical consequences; severe instances show notable rates of neonatal death [2]. The degree of the prognosis depends much on the size of the lungs at birth. Determining whether the liver is present in the chest and also the lung-to-head ratio (LHR) will help one ascertain this [3]. Although neonatal treatment has advanced, cases of severe CDH, which is defined by a liver placed in the chest and a low LHR, usually show a notable morbidity and mortality rate. A significant number of disorders affecting the newborn population are caused by the lungs' small size and their inherent malfunctioning nature. Some writers believe that a fetal lung heart rate less than one paired with the presence of a liver in the chest can be used to forecast the presence of hypoplasia, a very aberrant and maybe fatal lung function and size [4]. Thanks to improved understanding of the disease process and subsequent advances in care, some centers have seen newborn survival rates rise to as high as 90% [5].

Still, survival rates in most institutions range from 50% to 70%. Attaining stabilization and then proceeding with open repair during postnatal management requires access to a neonatal intensive care unit (NICU). Extracorporeal membrane oxygenation (ECMO) is used in some intensive care units when the technology is available. ECMO is a specialized technique of respiratory support in which blood is passed through an artificial lung [5]. Some facilities lack access to this technology. The fundamental objectives of postnatal care are to maintain cardiovascular stability, reduce general morbidity, and enhance oxygenation and ventilation while minimizing ventilator-induced lung damage, thereby reducing the risk of ventilator-induced lung damage. Maintaining cardiovascular stability is another aim [6].

Description of the intervention

In 1946, Gross was the first individual to delineate the surgical rectification of CDH within the neonatal period [7]. The objective of this surgery is to reduce the herniated abdominal contents and repair the diaphragmatic defect. A patch may be necessary, or it may not be; the issue can be resolved without it. Despite little alteration in core mending approaches over the past two decades, recent years have witnessed some promising advancements. These advancements encompass the use of contemporary materials for patch repair, as well as the application of laparoscopic and thoracoscopic (minimally invasive surgical) repair techniques [8]. The timing of repairs is currently tailored to facilitate an initial period of stability. This is undertaken in recognition of the potential hazards associated with quick repair in an unstable baby.

Advancements in prenatal imaging have enabled the detection of the majority of congenital heart disorders during the prenatal phase. The objective of prenatal surgical interventions and proven methodologies is to enhance lung size and function (pulmonary hypoplasia) during gestation, thereby improving lung function and ultimately enhancing the prognosis for the infant [9]. The primary factors contributing to a poor baby prognosis are pulmonary hypoplasia and pulmonary hypertension; however, intrauterine correction of diaphragmatic hernia, which involves repositioning the colon and liver into the belly, has demonstrated the capacity to ameliorate these diseases. This was shown by the results of the initial experimental investigation using animals [10].

Initially, prenatal repair was attempted via hysterotomy and fetal surgery; however, this approach is linked to increased maternal morbidity and is currently employed less frequently. Further study on animals indicated that blocking the fetal trachea stimulates lung growth and enhances lung development and function [11].

In 2004, a group of European researchers reported the initial outcomes of an intrauterine fetoscopic technique utilizing a reversible balloon device to occlude the trachea in fetuses with severe congenital cardiac abnormalities [12]. This organization was referred to as the fetoscopic endoluminal tracheal occlusion (FETO) task group, which is responsible for this procedure. FETO has become a prenatal technique of choice for increasing lung expansion in fetuses identified with severe CDH. The method involves passing a balloon by an endoscopic approach into the fetus's trachea. This blocks the airways, allowing fluid to build up in the lungs. The retention of fluid creates pressure, which in turn accelerates lung development and promotes their expansion accordingly. Once the balloon has been inflated before delivery, it must be removed to restore normal patency of the airway. It is predicated on the idea that tracheal closure reduces the outflow of lung fluid, therefore raising intrapulmonary pressure and encouraging the growth of lung tissue. The foundation of the justification for FETO technology is this knowledge. Research on animals has revealed that this approach can enhance lung development, providing a basis for its application in the early stages of human fetal development [13]. Clinical research findings have been conflicting; some highlight rising survival rates while others draw attention to possible risks and challenges related to the operation [14].

Importance of this review

The care of severe CDH is still difficult, even with developments in prenatal imaging and surgical methods. Still under research is the effect of FETO in changing the usual course of the disease [15]. The occurrence of conflicting results in previous studies regarding the effectiveness and safety of FETO emphasizes the need for a thorough investigation of the currently available evidence. This systematic study aims to evaluate the impact of FETO on outcomes compared to conventional postnatal therapy for mothers and children, thereby providing informed recommendations for treatment choices for severe CDH to doctors and patients [16].

## Review

Materials and methods

This systematic review aimed to assess the effectiveness and safety of prenatal intervention, specifically FETO, compared to postnatal management for CDH in improving fetal and maternal outcomes. The target population included pregnant women carrying fetuses with CDH, encompassing both severe and moderate cases. The intervention under review was FETO, while the comparison group received expectant management with postnatal surgical repair. Outcomes of interest included neonatal survival and morbidity, such as respiratory support needs, pulmonary hypertension, gastrointestinal and airway complications, and maternal outcomes, including the risk of preterm premature rupture of membranes (PPROM), preterm labor, and infection.

Studies were included if they reported on prenatal intervention for CDH, specifically FETO, and provided data on fetal and/or maternal outcomes. Studies were excluded if they did not report primary data, were case reports, or were not available in English. A comprehensive literature search was conducted using PubMed, OpenAlex, and CENTRAL databases. The search strategy employed the following string across all databases: (Hemidiaphragm Agenes* OR Diaphragm Unilateral Agenes* OR Congenital Diaphragmatic Defect* OR Congenital Diaphragmatic Hernia* OR Morgagn* Hernia* OR Bochdalek Hernia*) AND (Fetoscop* OR Amnioscopic Surgical Procedure* OR Amnioscopic Surger* OR Amnioscop* OR Embryoscopic Surgical Procedure* OR Embryoscopic Surger* OR Embryoscop* OR Fetoscopic Surgical Procedure* OR Fetoscopic Surger*). An alternative strategy using Medical Subject Headings (MeSH) was also applied: ("Hernias, Diaphragmatic, Congenital/surgery" (MeSH) OR "Hernias, Diaphragmatic, Congenital/therapy" (MeSH)).

**Figure 1:**
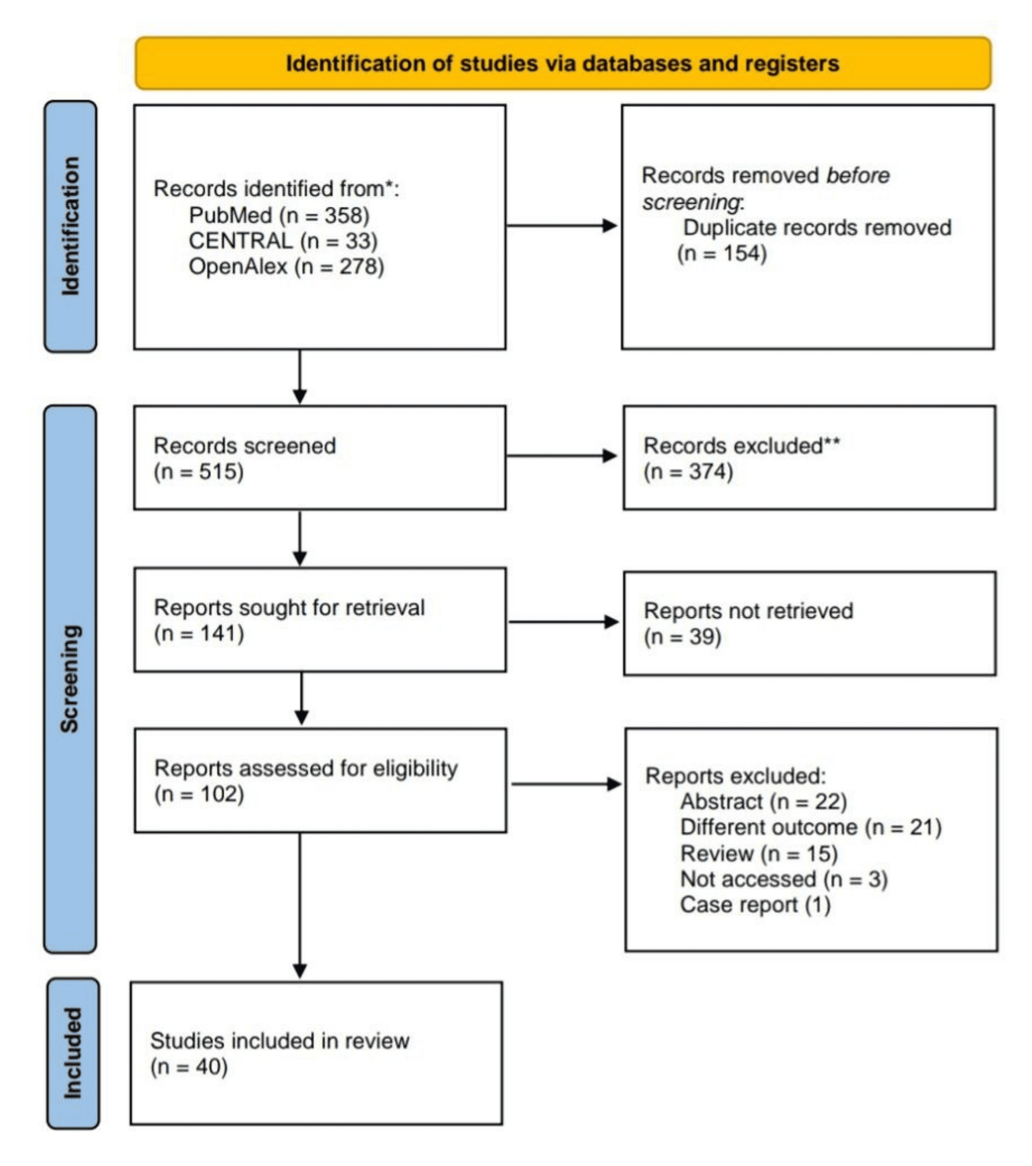
PRISMA flow diagram PRISMA: Preferred Reporting Items for Systematic Reviews and Meta-Analyses [17]

All identified records were screened for duplicates and assessed for relevance. Initial screening was conducted on titles and abstracts, followed by full-text review of potentially eligible studies. Data were then extracted systematically using predefined criteria. Figure 1 shows the Preferred Reporting Items for Systematic Reviews and Meta-Analyses (PRISMA) flow diagram.

Results

Literature Search Results

The database search (PubMed, Central, and OpenAlex) initially identified 669 papers. After removing duplicates and irrelevant articles, 141 papers were eligible for screening. Following a screening of titles and abstracts, 102 studies were sought for full-text retrieval. Finally, after full-text screening with the application of inclusion and exclusion criteria, 40 studies were included in the review.

Types of Studies

The review examined 40 studies that investigated prenatal interventions for CDH. The top evidence came from four RCTs assigning participants to either FETO or a control group, typically expectant management. Additionally, 11 were prospective cohort studies, which followed pregnant women over time and observed outcomes in both FETO and expectant management groups. Retrospective cohort studies were 14. Furthermore, we included three studies that focused on describing FETO outcomes without a direct comparison group and two studies that performed a reanalysis of pooled individual participant data from multiple multicenter RCTs. Finally, six studies were categorized as prospective controlled studies or single-arm clinical trials.

Types of Participants

The participants were pregnant women carrying fetuses diagnosed with CDH. Many studies have concentrated on severe cases, characterized by liver herniation, a low LHR, or an anticipated need for ECMO. The side of the CDH defect (left-sided or right-sided) was also considered, with some research directly comparing outcomes between these groups.

Types of Interventions

FETO was the primary prenatal intervention studied. It is a minimally invasive procedure and involves temporarily occluding the fetal trachea with a detachable balloon inserted fetoscopically to stimulate lung growth. Studies investigating the timing of FETO compared "early" intervention (22-24 weeks of gestation) to "standard" timing (26-30 weeks). Some research has also examined the use of a collagen plug to seal the fetoscopic access site in the fetal membranes, aiming to reduce the risk of PPROM. Control groups typically received expectant management, involving standard prenatal and postnatal care. In addition to FETO, some studies considered other interventions, such as postnatal ECMO for respiratory support and surfactant therapy to improve lung function. One study compared outcomes across groups receiving FETO, ECMO, or neither.

Outcome Measures

We assessed both neonatal and maternal outcomes. Neonatal outcomes included survival at various time points (e.g., 7 days, 28 days, 90 days, 6 months, and survival to hospital discharge). Morbidity outcomes focused mainly on respiratory support needs (ECMO, ventilation, oxygen), pulmonary hypertension, gastrointestinal complications, and airway issues (tracheomalacia, tracheomegaly). Maternal outcomes primarily included the risk of PPROM, preterm labor, and infection (Tables 1-4).

**Table 1:** Summary of the included studies FETO: fetoscopic endoluminal tracheal occlusion, CDH: congenital diaphragmatic hernia, LHR: lung-to-head ratio, LCDH: left-sided congenital diaphragmatic hernia, N/A: not applicable, RCT: randomized controlled trial, ECMO: extracorporeal membrane oxygenation, TTTS: twin-to-twin transfusion syndrome, RCDH: right-sided congenital diaphragmatic hernia, O/E LHR: observed/expected lung-to-head ratio

Year, author	Study design	Main interventions	Intervention group	Control group
2003, Harrison et al. [9]	RCT	FETO using a detachable silicone balloon placed in the fetal trachea via bronchoscopy	The intervention group received FETO	The control group received standard postnatal care (expectant management with planned delivery at 36 weeks and intensive neonatal care at a tertiary center)
2005, Cortes et al. [18]	Single-center, randomized, controlled clinical trial	(1) FETO, (2) standard perinatal care (control)	The main intervention group received FETO	The control group received standard perinatal care without FETO
2011, Deprest et al. [12]	Retrospective multicenter study with a prospective validation component.	FETO	Fetuses with severe isolated CDH (LCDH or RCDH) with liver herniation and a LHR <1.0 who underwent FETO	The control group comprised 2 sets: (1) a retrospective cohort of 134 patients with isolated LCDH who did not undergo FETO, and (2) a prospective cohort of 53 patients with isolated CDH seen before 28 weeks of gestation, who either received standard postnatal care or had their pregnancy terminated
2006, Jani et al. [13]	Combination of a prospective, consecutive case series and a retrospective multicenter review	FETO	24 fetuses with severe LCDH who underwent FETO between 26 and 28 weeks of gestation	The retrospective validation study implicitly defines the control group. It consists of 37 fetuses with isolated LCDH who met the selection criteria for poor prognosis but did not undergo FETO
2007, Saura et al. [19]	Retrospective cohort study	FETO using a balloon	13 fetuses diagnosed with CDH who underwent FETO before birth because they had an LHR <1.1	6 fetuses diagnosed with CDH who did not undergo FETO. 3 had an LHR ≥1.4, and 3 were not seen prenatally
2006, Jani et al. [20]	Multicenter prospective and ongoing study	FETO	Singleton pregnancies with severe CDH, diagnosed by sonographic evidence of intrathoracic liver herniation and a low LHR (O/E LHR ≤ 1), treated with FETO	N/A
2012, Ruano et al. [14]	Prospective controlled study	(1) FETO, (2) prenatal expectant management	The main intervention group is the FETO group, where fetuses underwent FETO using a 1.0-mm fetoscope	The control group received no prenatal intervention and underwent standard postnatal care
2011, Peralta et al. [21]	Prospective study	FETO	8 fetuses with severe isolated LCDH and normal karyotypes who underwent FETO. Initially, 13 fetuses underwent FETO, but 5 were excluded from the final analysis due to various reasons (RCDH, maternal decision to interrupt treatment, and postnatal diagnosis of trisomy 13)	N/A
2012, Ruano et al. [22]	RCT	(1) FETO, (2) postnatal management	The main intervention group received FETO	The control group received standard postnatal management without any fetal intervention
2013, Ruano et al. [23]	Multicenter study comparing early FETO with standard FETO and a control group with no intervention	(1) Early FETO performed between 22 and 24 weeks' gestation, (2) standard FETO performed between 26 and 30 weeks' gestation, (3) control: no FETO	The early FETO group, where fetuses underwent FETO between 22 and 24 weeks' gestation	10 fetuses with extremely severe isolated CDH who received no fetoscopic intervention (prenatal expectant management)
2013, Ali et al. [24]	Retrospective review	FETO	Infants with CDH who underwent FETO	N/A
2013, Doné et al. [25]	Prospective, multicenter study	FETO	Fetuses with severe isolated CDH who underwent FETO	Fetuses with either severe or moderate CDH who were managed expectantly (without FETO)
2014, Engels et al. [26]	Single-center cohort study	The use of a collagen plug to seal the fetal membrane defect after FETO for CDH	Patients who received a collagen plug to seal the fetal membrane defect after FETO	Patients who underwent FETO but did not receive a collagen plug
2017, Persico et al. [27]	Retrospective, single-center cohort study	FETO	21 fetuses with severe CDH who underwent FETO	N/A
2017, Braga et al. [28]	Retrospective, descriptive study	FETO using an inflatable balloon	Fetuses who had severe CDH and underwent FETO	N/A
2016, Ali et al. [29]	Retrospective review	FETO	Infants who underwent FETO	Infants with CDH who did not undergo FETO
2018, Kosinski et al. [30]	Prospective study	FETO	Singleton pregnancies with isolated LCDH and an observed over expected lung area to head circumference ratio (o/e LHR) <25%, treated with FETO	N/A
2017, Jiménez et al. [31]	Retrospective analysis of prospectively collected data from 3 centers	FETO using a balloon to occlude the fetal trachea	Fetuses with CDH who underwent FETO followed by balloon removal	N/A
2017, Belfort et al. [32]	Prospective observational cohort study comparing outcomes with a historical control cohort	FETO	Fetuses diagnosed with severe LCDH (LHR ≤1.0 and liver herniation) who underwent FETO	A historical cohort of pregnant women with fetuses diagnosed with severe LCDH (similar severity criteria as the intervention group) who did not undergo FETO
2018, Snoek et al. [33]	Observational cohort study	(1) ECMO used to support infants with severe respiratory failure, (2) FETO, (3) standardized postnatal management	The study compares outcomes across different groups receiving ECMO, FETO, or neither, within the context of a standardized postnatal management protocol	The authors compare outcomes across groups receiving different treatments (ECMO, FETO) and those receiving only the standardized postnatal management
2019, Style et al. [34]	Retrospective cohort study	FETO	Fetuses with severe CDH who underwent FETO	Fetuses with severe CDH who did not undergo FETO
2024, Ali et al. [35]	Retrospective review	FETO	Infants with RCDH or LCDH who underwent FETO	Infants with CDH who did not undergo FETO. The study also compares outcomes between RCDH and LCDH within both the FETO and non-FETO groups
2020, Baschat et al. [36]	Prospective cohort study	FETO	Fetuses diagnosed with severe CDH who underwent FETO	N/A
2020, Wada et al. [37]	Single-arm clinical trial	FETO	Fetuses with severe isolated LCDH meeting specific criteria (liver herniation, Kitano grade 3 stomach position, o/e LHR <45%, no other anomalies, normal karyotype, cervical length >20 mm, no hypertensive disorders). These fetuses underwent FETO	N/A
2020, Cruz-Martínez et al. [38]	Prospective cohort study with individual matching of cases and controls	FETO	Fetuses with isolated LCDH, normal karyotype, and severe pulmonary hypoplasia (O/E LHR <26% and liver herniation) who underwent FETO before 32 weeks of gestation	Fetuses with similar characteristics (isolated LCDH, normal karyotype, and similar O/E LHR values ±2%) who were managed expectantly during pregnancy (no FETO)
2021, Deprest et al. [39]	Multicenter, open-label, RCT	(1) FETO at 27 to 29 weeks of gestation, followed by standardized postnatal care, or (2) expectant care, followed by standardized postnatal care	The main intervention group received FETO at 27 to 29 weeks of gestation	The control group received expectant care
2021, Deprest et al. [40]	Multicenter, open-label, adaptive, parallel-group, superiority RCT	(1) FETO, (2) expectant care	The FETO group underwent FETO at 30 to 32 weeks of gestation	The expectant group received standard prenatal and postnatal care without fetal surgery
2022, Trad et al. [41]	Retrospective cohort study	(1) Ultrasound-guided procedures, (2) fetoscopic procedures including FETO for CDH, (3) open procedures	The study compares 3 groups of interventions (ultrasound-guided, fetoscopic, and open) to each other. The largest group was the fetoscopic procedures, but this is due to the prevalence of TTTS cases, not a designation of a "main" group	N/A
2022, Donepudi et al. [42]	Retrospective study	FETO	Fetuses who underwent FETO	Fetuses with moderate CDH who did not undergo FETO (non-FETO cohort)
2022, Cruz-Martínez et al. [43]	Prospective, matched cohort study	FETO	29 fetuses with LCDH and moderate lung hypoplasia who underwent FETO	29 fetuses with LCDH and moderate lung hypoplasia who did not undergo FETO and received standard postnatal care
2021, Russo et al. [44]	Retrospective cohort study	Expectant management and FETO	Fetuses with isolated RCDH that underwent FETO	Fetuses managed expectantly (without FETO)
2022, Van Calster et al. [45]	Reanalysis of pooled individual participant data from 2 multicenter RCTs	FETO with expectant prenatal management	The main intervention group received FETO	The control group received expectant prenatal management
2023, Sferra et al. [46]	Prospective study comparing a cohort of severe CDH patients undergoing FETO to a cohort of non-FETO CDH patients with severe disease	FETO	Children with severe CDH who underwent FETO	A cohort of non-FETO CDH patients with severe disease, defined by liver herniation, large defect size, and/or ECMO use
2024, Wild et al. [47]	Retrospective single-center cohort study	FETO	Infants treated with FETO	Infants who met FETO inclusion criteria but did not receive FETO and instead received SOC
2024, Bergh et al. [48]	Multicenter, retrospective cohort study with a comparative analysis of FETO vs. expectant management	FETO	Patients with isolated, severe, or moderate LCDH who underwent FETO	Patients with isolated, severe, or moderate LCDH who received expectant management
2024, Dütemeyer et al. [49]	Multicenter, retrospective study	(1) FETO, (2) expectant management: this group frequently utilized ECMO postnatally	Fetuses that underwent FETO	The control group is the fetuses who received expectant management
2024, Sevilmis et al. [50]	Single-center retrospective cohort review	The use of surfactant therapy in infants with CDH	The CDH infants who received surfactant. A subgroup of this group also received FETO prenatally	The control group consists of CDH infants who did not receive surfactant. This group is further subdivided into those who underwent FETO and those who did not. A further control group is created by selecting only the non-FETO patients with severe CDH defects to compare to the FETO group
2024, Sferra et al. [51]	Prospective study	FETO	Severe CDH patients undergoing FETO	Severe CDH patients who did not undergo FETO (expectantly managed). This group was further stratified by disease severity into high, medium, and low severity groups
2024, Manfroi et al. [52]	Retrospective cohort study	FETO	Singleton pregnancies with fetal CDH treated with FETO. Specifically, 46 fetuses met the inclusion criteria and underwent the procedure	N/A
2024, Olutoye et al. [53]	Single-center retrospective cohort review	FETO	CDH patients who underwent FETO	N/A

**Table 2:** Survival outcomes among the neonates of the intervention and/or control groups FETO: fetoscopic endoluminal tracheal occlusion, CDH: congenital diaphragmatic hernia, N/A: not applicable, LCDH: left-sided congenital diaphragmatic hernia, RCDH: right-sided congenital diaphragmatic hernia, LHR: lung-to-head ratio, ECMO: extracorporeal membrane oxygenation, CI: confidence interval, NICU: neonatal intensive care unit, O/E LHR: observed/expected lung-to-head ratio

Year, author	Neonatal survival outcome/s among the intervention group	Neonatal survival outcome/s among the control group
2003, Harrison et al. [9]	8 out of 11 fetuses (73%) survived to 90 days of age	10 out of 13 fetuses (77%) survived to 90 days of age
2005, Cortes et al. [18]	73% survival at 90 days. 1 late death occurred from chronic pulmonary hypertension	77% survival at 90 days. 1 late death occurred from chronic pulmonary hypertension
2011, Deprest et al. [12]	In the retrospective analysis of 20 FETO patients, 50% survived to discharge. In the prospective validation, of the 12 patients who did not undergo FETO and received standard postnatal care, only 1 (8.3%) survived	In the retrospective control group (134 patients), overall survival to discharge was 47% (58/123) in liveborn babies and 43% (58/134) in all antenatally diagnosed cases. In the prospective control group (53 patients), overall survival to discharge was 52.3% (23/44) in liveborn babies and 43.4% (23/53) in all antenatally diagnosed cases. Among the 12 patients in the prospective group who met FETO criteria but did not receive it, only 1 survived
2006, Jani et al. [13]	Among the 24 fetuses in the intervention group, early (7-day) survival was 75% (18 of 24), late (28-day) survival was 58.3% (14 of 24), and survival to hospital discharge was 50% (12 of 24). This increases to 55% after excluding 2 deaths due to prenatally missed associated problems	In the validation study's control group of 37 fetuses, antenatal loss was 13% (5 of 37), and survival to discharge was 9% (3 of 32 live births). The retrospective analysis of 134 patients showed an overall survival rate of 43% (58 of 134) and 47% (58 of 123) in liveborn babies
2007, Saura et al. [19]	In the FETO group, 7 out of 13 (53.8%) survived to 6 months of age. Immediate mortality was 5/13 (38.46%), including those who didn't undergo surgery and the ECMO case. 1 additional infant died at 4 months of age after discharge	In the non-FETO group, 5 out of 6 (83.3%) survived. 1 patient died before surgery due to a severe cardiac malformation
2006, Jani et al. [20]	Of 210 fetuses undergoing FETO, 204 (97.1%) were liveborn, 98 (48.0%) survived and were discharged alive from the hospital, and 10 deaths were directly related to difficulties removing the balloon	There was no explicit control group. However, based on the antenatal CDH registry data, the estimated survival rate for expectantly managed fetuses with LCDH was 24.1%, and 0% for RCDH
2012, Ruano et al. [14]	Nine out of 17 (52.9%) infants in the FETO group survived up to 28 days after birth. In the intention-to-treat analysis (including the case where FETO failed due to placental bleeding), 9 of 17 (52.9%) survived. Excluding the unsuccessful FETO case, 9 of 16 (56.3%) survived	1 out of 18 (5.6%) infants in the control group survived up to 28 days after birth
2011, Peralta et al. [21]	Among the 8 fetuses in the final analysis, 4 (50%) survived to hospital discharge. The remaining 4 died within 24 hours of birth due to severe lung hypoplasia and/or sepsis	N/A
2012, Ruano et al. [22]	In the intention-to-treat analysis, 10 out of 20 (50%) infants in the FETO group survived to 6 months. In the received-treatment analysis (excluding those who declined treatment), 10 out of 19 (52.6%) survived	In the intention-to-treat analysis, 1 out of 21 (4.8%) infants in the control group survived to 6 months. In the received-treatment analysis, 1 out of 19 (5.3%) survived
2013, Ruano et al. [23]	A survival rate of 62.5% (5 out of 8 infants) to 180 days (6 months)	A survival rate of 0% (0 out of 10 infants)
2013, Ali et al. [24]	The overall survival rate was 48%. Survival was significantly lower in infants born prematurely (43% vs. 70% for those born at term), and particularly low for those born before 35 weeks (18% vs. 82% for those born at or after 35 weeks). No infants born before 33 weeks survived	The authors cite previous studies reporting survival rates of 60-70% for non-FETO CDH infants, 64% for term births, and 35% for premature births in 1 study, and 73.1% and 53.5%, respectively, in another
2013, Doné et al. [25]	Of 188 fetuses with isolated CDH who underwent FETO, 90 (49.2%) survived to discharge. Early neonatal survival (7 days) was 63.9%, and late neonatal survival (28 days) was 55.7%	The control group data came from a previous study: among fetuses with severe hypoplasia, only 4 survived to discharge. Among those with moderate hypoplasia, 37 survived to discharge
2014, Engels et al. [26]	In the collagen plug group, the survival rate at 7 days was 54%, and the survival rate at discharge was 46%	In the no-collagen plug group, the survival rate at 7 days was 51%, and the survival rate at discharge was 38%
2017, Persico et al. [27]	Overall postnatal survival at 1–3 years of age in the 21 cases was 38.1% (8/21). In the subgroup of 17 cases with isolated unilateral CDH, survival was 47.1% (8/17)	The authors cite a previous study with an expectant management survival rate of 4.8%
2017, Braga et al. [28]	Of the 22 fetuses that underwent successful FETO, 15 (68%) survived after surgery. Overall, considering the 28 cases (including the 6 unsuccessful FETO attempts), 17 newborns (60.7%) survived	There was no true control group. However, the authors refer to the 6 cases where FETO failed. In this group, 1 fetus died in utero, 3 died after birth without CDH repair, and 2 survived after surgery
2016, Ali et al. [29]	The mortality rate in the FETO group was not significantly different from the non-FETO group (p=0.30). 5 infants died in the labor suite and could not be resuscitated	The mortality rate in the non-FETO group was not significantly different from the FETO group (p=0.30)
2018, Kosinski et al. [30]	A neonatal survival rate of 46.4% (13/28) at the time of discharge	N/A
2017, Jiménez et al. [31]	The study reports that all balloons were eventually successfully removed in the 302 in-house procedures. However, in nine cases where balloon removal was attempted outside the FETO centers, 3 failed, resulting in neonatal death. 1 additional fetal death occurred 10 days after a successful fetoscopic balloon removal, but the cause seemed likely unrelated to the procedure	The authors refer to historical control data showing improved survival rates with FETO compared to historical controls (24% to 49% in LCDH, and 17% to 42% in RCDH with observed/expected LHR <45%)
2017, Belfort et al. [32]	6-month survival: 80% (8/10), 1-year survival: 70% (7/10), 2-year survival: 67% (6/9), survival to discharge: 67% (6/9), survival to date: 70% (7/10)	6-month survival: 11% (1/9), 1-year survival: 11% (1/9), 2-year survival: 11% (1/9)
2018, Snoek et al. [33]	The survival rates varied significantly depending on the intervention and the center. For ECMO, survival rates ranged from 40.3% (Rotterdam) to 78.1% (Mannheim). For FETO, 52.4% of patients survived. Overall, 71.9% of the total cohort survived	There is no true control group. The survival rate for those not receiving ECMO or FETO is implied by the overall survival rate (71.9%) and the survival rates of the ECMO and FETO groups
2019, Style et al. [34]	In the FETO group with severe CDH, survival at discharge was 81.3% (13/16). Survival at 6 months and 1 year was 75% (12/16) and 75% (12/16), respectively	In the non-FETO group with severe CDH, survival at discharge was 60% (15/25). Survival at 6 months and 1 year was 64% (16/25) and 64% (16/25), respectively
2024, Ali et al. [35]	Among infants who underwent FETO, there was no statistically significant difference in survival between those with RCDH and LCDH (67% vs. 51%, p=0.403)	Overall survival was 60% for LCDH and 50% for RCDH (p=0.375)
2020, Baschat et al. [36]	Neonatal survival at 28 days was 93% (95% CI 49–100%), and survival to 6 months or hospital discharge was 86% (95% CI 44–100%)	N/A
2020, Wada et al. [37]	The survival rate at 90 days of age and the survival rate to discharge were both 45% (5/11). 1 fetal death occurred at 33 weeks of gestation due to cord strangulation from a detached amniotic membrane	N/A
2020, Cruz-Martínez et al. [38]	32% survival rate up to 28 days after birth	0% survival rate up to 28 days after birth
2021, Deprest et al. [39]	Survival to discharge from the NICU: 63% (62 of 98 infants) in the intention-to-treat analysis; 66% (57/88) in the per-protocol analysis. Survival to 6 months without oxygen supplementation: 54% (53 of 98 infants) in the intention-to-treat analysis; 57% (51/88) in the per-protocol analysis	Survival to discharge from the NICU: 50% (49 of 98 infants) in the intention-to-treat analysis; 51% (48/95) in the per-protocol analysis. Survival to 6 months without oxygen supplementation: 44% (43 of 98 infants) in the intention-to-treat analysis; 45% (43/95) in the per-protocol analysis
2021, Deprest et al. [40]	In the intention-to-treat analysis, 40% (16 of 40) of infants in the FETO group survived to discharge from the NICU. This was sustained to 6 months of age. In a post-hoc analysis that included additional participants, the survival rate to discharge was 36%	In the intention-to-treat analysis, 15% (6 of 40) of infants in the expectant care group survived to discharge from the NICU. This was sustained to 6 months of age. In a post-hoc analysis that included additional participants, the survival rate to discharge was 14%
2022, Donepudi et al. [42]	77.8% (7/9) survival to discharge; 66.7% (6/9) survival at 1 year	57.1% (12/21) survival to discharge; 57.1% (12/21) survival at 1 year
2022, Cruz-Martínez et al. [43]	In the FETO group: 51.7% survival at 28 days, 48.3% survival at discharge, 41.4% survival at 6 months	In the control group: 24.1% survival at 28 days, 24.1% survival at discharge, 24.1% survival at 6 months
2021, Russo et al. [44]	In the FETO group, overall survival at discharge was 41% in fetuses with O/E LHR <45%. Survival was higher with FETO compared to expectant management in fetuses with similar lung size (41% vs. 15%). Survival at 6 and 12 months was also significantly increased with FETO compared to expectant management	In the expectant management group, overall survival was 46%. Survival rate was significantly lower in those with O/E LHR <45% (15%) compared to those with O/E LHR ≥45% (61%). None of the fetuses with O/E LHR <30% survived. Survival at 6 and 12 months was also significantly lower in those with O/E LHR <45%
2022, Van Calster et al. [45]	In the FETO group, survival to discharge from the NICU was 54%, survival to 6 months was 54%, and survival to 6 months without oxygen supplementation was 43%	In the expectant management group, survival to discharge from the NICU was 39%, survival to 6 months was 39%, and survival to 6 months without oxygen supplementation was 32%
2023, Sferra et al. [46]	78% survival rate at discharge and 67% survival rate at 5 years	59% survival rate at discharge and 59% survival rate at 5 years
2024, Wild et al. [47]	91.7% survival rate	71.4% survival rate
2024, Bergh et al. [48]	6-month survival rate was 69.8%. While higher than the control group, but was not significant (p=0.30)	6-month survival rate was 58.1%
2024, Dütemeyer et al. [49]	In the FETO group: survival rate at discharge: 44.7%, survival rate at 2 years of age: 42.5%	In the expectant management group: survival rate at discharge: 74.3%, survival rate at 2 years of age: 72.8%
2024, Sevilmis et al. [50]	Overall, 8/25 (32%) of surfactant recipients did not survive to discharge. However, survival to discharge was higher (92%) among FETO patients who received surfactant compared to those who did not (86%). The overall 1-year mortality rate was 20% (21/105)	Among the non-FETO patients who did not receive surfactant, 85% survived to discharge. Among the FETO patients who did not receive surfactant, 86% survived to discharge. The overall 1-year survival rate was 83% (87/105)
2024, Sferra et al. [51]	The study reports a 78% survival rate to discharge and a 67% long-term survival rate at a median follow-up of more than five years for the FETO group	The 6-month survival rate for severe CDH patients in the TOTAL trial was 40%
2024, Manfroi et al. [52]	Infant survival rates were 37% at 28 days and 34.8% at six months	N/A
2024, Olutoye et al. [53]	23 out of 34 (70%) patients survived to discharge	N/A

**Table 3:** Morbidity outcomes among the neonates in the intervention and/or control groups FETO: fetoscopic endoluminal tracheal occlusion, PPROM: preterm premature rupture of membrane, N/A: not applicable, CDH: congenital diaphragmatic hernia, ECMO: extracorporeal membrane oxygenation, APGAR: appearance, pulse, grimace, activity, and respiration, HFOV: high-frequency oscillatory ventilation, iNO: inhaled nitric oxide, EXIT: ex utero intrapartum treatment, NICU: neonatal intensive care unit, PH: pulmonary hypertension, O/E LHR: observed/expected lung-to-head ratio, GERD: gastroesophageal reflux disease, SOC: standard of care, O/E TFLV: observed/expected total fetal lung volume, CLAMS: clinical linguistic and auditory milestone scale, DQ: developmental quotient, CAT: cognitive adaptive test, ECLS: extracorporeal life support, ENT: ear, nose, and throat, LCDH: left-sided congenital diaphragmatic hernia, RCDH: right-sided congenital diaphragmatic hernia

Year, author	Neonatal morbid outcome/s among the intervention group	Neonatal morbid outcome/s among the control group
2003, Harrison et al. [9]	All infants required intensive respiratory support. 2 infants died shortly after repair (1 from respiratory insufficiency at 9 days, and the other from sepsis at 14 days); a third died at 71 days from PH. There was substantial gastrointestinal morbidity, with no significant difference compared to the control group in rates of respiratory and gastrointestinal complications, age at repair, need for prosthetic patch, age at extubation, age at discharge, or need for supplemental oxygen. 5 of 8 survivors had evidence of mild-to-moderate white-matter injury on brain imaging	All infants required intensive respiratory support. 1 infant received 5 days of ECMO before repair. 3 infants died (2 before repair, 1 from respiratory insufficiency and another from previously undiagnosed Fryns syndrome; 1 after repair from PH). There was substantial gastrointestinal morbidity, with no significant difference compared to the intervention group in rates of respiratory and gastrointestinal complications, age at repair, need for prosthetic patch, age at extubation, age at discharge, or need for supplemental oxygen. 3 of 9 survivors had evidence of mild-to-moderate white-matter injury on brain imaging
2005, Cortes et al. [18]	The TO group experienced significant prematurity, severe growth failure at 1 year (86%), moderate growth failure at 2 years (33%), a high rate (43%) of recurrent herniations, and 43% required supplemental oxygen at discharge. 43% also had hearing loss requiring amplification	The control group experienced severe growth failure at 1 year (56%), moderate growth failure at 2 years (22%), a high rate (67%) of recurrent herniations, and 44% required supplemental oxygen at discharge. 44% also had hearing loss requiring amplification
2011, Deprest et al. [12]	In the FETO group, 8 babies died in the neonatal period due to complications of pulmonary hypoplasia. 2 additional nonsurvivors died from other causes (deep venous thrombosis and liver failure associated with a chromosomal abnormality). The long-term survivors (10 babies) showed no apparent neurologic morbidity at a median follow-up of 19 months	In the retrospective control group, the cause of death for those who did not survive was not explicitly stated. In the prospective control group, 11 of 12 babies who did not receive FETO died in the neonatal period due to pulmonary hypoplasia and hypertension
2006, Jani et al. [13]	In the intervention group, morbidities included: pulmonary hypoplasia and hypertension (6 of 12 nonsurvivors), preterm delivery and PPROM (4 of 12 nonsurvivors), balloon dislodgement (2 of 12 nonsurvivors), and other non-pulmonary complications (3 of 12 nonsurvivors; including chromosomal abnormality, catheter complication, respiratory infection, and chylothorax). 4 babies required prolonged oxygen support after discharge	N/A
2007, Saura et al. [19]	In the FETO group, early complications included 1 reherniation (11.1%) and 2 intestinal occlusions (22.2%)	In the non-FETO group, early complications included 1 reherniation and 1 hiatus hernia. A late complication was 1 reherniation at 5 months of age. 1 patient required oxygen at home
2006, Jani et al. [20]	Neonatal deaths (106 cases) were mainly due to pulmonary hypoplasia, PH, and prematurity. High rate of preterm delivery (30.9% before 34 weeks)	N/A
2011, Peralta et al. [21]	Among the 4 surviving neonates, 1 required oxygen support at home for 1 month, 1 developed intestinal obstruction requiring surgery, and 1 presented with gastroesophageal reflux. 2 of the 4 survivors required cardiovascular drugs and iNO due to severe PH. The median total time of mechanical ventilation was 16.5 days (range: 8–35), and the median total time spent in the NICU was 22.5 days (range: 10–44)	N/A
2012, Ruano et al. [14]	8 out of 17 (47.1%) infants in the FETO group experienced severe pulmonary arterial hypertension. 1 infant died due to postsurgical infection after CDH repair. 1 infant died due to secondary infection from prolonged parenteral nutrition. In 1 case, selective right bronchial occlusion occurred during the FETO procedure, resulting in death	Sixteen out of 18 (88.9%) infants in the control group experienced severe pulmonary arterial hypertension
2012, Ruano et al. [22]	In the FETO group, 1 infant died after postnatal surgical repair due to heart failure from severe PH, and another died from aspiration pneumonia due to megaesophagus and severe esophageal reflux. Severe pulmonary arterial hypertension occurred in 10 (50%) of the infants	In the control group, 2 infants died after surgery due to heart failure related to severe PH, and 1 died from pneumonia. Severe pulmonary arterial hypertension occurred in 18 (85.7%) of the infants
2013, Ruano et al. [23]	25% (2 out of 8 infants) experienced severe postnatal pulmonary arterial hypertension. 75% (6 out of 8) required postnatal surgical repair, all of which used a prosthetic patch. 2 infants with prolonged tracheal occlusion (over 11 weeks) developed tracheomegaly; 1 died due to ventilation difficulties	90% (9 out of 10 infants) experienced severe postnatal pulmonary arterial hypertension. 20% (2 out of 10) required postnatal surgical repair, both using a prosthetic patch
2013, Doné et al. [25]	Among the 90 FETO survivors: assisted ventilation lasted a median of 15 days; 48% needed oxygen for at least 28 days; 27% experienced PH; surgery was performed at a median age of 2 days; 77% required patch repair; full enteral feeding was achieved at a median age of 26 days; 10% required fundoplication; and 52% received antacids	Data from the previous study showed that among the 4 survivors with severe hypoplasia, morbidity was uniformly worse than in the FETO group (except for patch repair). Among the 37 survivors with moderate hypoplasia, the median duration of assisted ventilation was 19 days, 27% needed oxygen at 28 days, and 30% experienced PH
2013, Ali et al. [24]	Infants born before 35 weeks required longer ventilation (median 45 vs. 12 days), had a higher rate of surgery for gastroesophageal reflux (50% vs. 9%), and had lower observed: expected LHR. Other morbidities included tracheomalacia (in 6 of 29 survivors), upper airway symptoms (6 of 29 survivors), hernia recurrence (7 of 29 survivors), growth failure (7 of 28 survivors), and delayed motor milestones (1 of 28 survivors)	N/A
2017, Persico et al. [27]	Causes of death included severe PH (7 cases) and cardiorespiratory arrest (2 cases). Suprasystemic PH within 24 hours of birth was observed in all neonates who died and in 50% of survivors. 1 survivor was oxygen-dependent at discharge and later died from PH. Postnatal surgical repair was required in most cases	N/A
2016, Ali et al. [29]	The FETO group experienced significantly longer durations of mechanical ventilation, supplementary oxygen, and hospital stay compared to the non-FETO group. They also had a higher rate of patch repair, indicating larger diaphragmatic defects	The non-FETO group had shorter durations of mechanical ventilation, supplementary oxygen, and hospital stay compared to the FETO group. They also had a lower rate of patch repair
2017, Jiménez et al. [31]	In 2 cases of endoscopic balloon retrieval, minor tracheal epithelial defects were observed during tracheoscopy, but these had no postnatal clinical consequences	The authors cite previous studies showing reduced morbidity rates with FETO compared to historical controls
2017, Belfort et al. [32]	Need for ECMO: 30% (3/10); supplemental oxygen at 6 months: 50% (4/8); prolonged intubation (median 33 days, range 11-241 days). 1 infant died at 17 days of life due to severe PH, another at 4 months due to PH and pulmonary capillary hemangiomatosis, and a third at 8 months due to persistent PH and respiratory failure	Need for ECMO: 78% (7/9); prolonged intubation (median 30 days, range 15-235 days). All but 1 infant died
2018, Kosinski et al. [30]	Neonatal death (14/28), primarily due to pulmonary hypoplasia and hypertension. Most deaths (8/14) occurred after neonatal surgery for CDH. PPROM occurred in 17/28 (61%) of cases, with 5/28 (18%) occurring before 34 weeks of gestation. Lower APGAR scores in the tenth minute were observed in non-survivors	N/A
2016, Ali et al. [29]	In the FETO group, there were no statistically significant differences in short-term outcomes (duration of ventilation, need for HFOV, iNO, time to full feeds, need for surgical repair, time to surgery, patch repair, postoperative mortality, length of stay) between RCDH and LCDH	In the non-FETO group, Infants with right-sided CDH had a significantly higher need for iNO (p=0.036). There were no significant differences in other short-term outcomes (duration of ventilation, HFOV, time to full feeds, surgical repair, time to surgery, patch repair, postoperative mortality, length of hospital stay) between RCDH and LCDH
2019, Style et al. [34]	Among neonates in the FETO group with severe CDH, 43.8% (7/16) required ECMO, and the median length of hospital stay was 78 days. At 6 months, 25% (3/12) required supplemental oxygen	In the non-FETO group with severe CDH, 84% (21/25) required ECMO, and the median length of hospital stay was 79 days. At 6 months, 62.5% (10/16) required supplemental oxygen
2020, Baschat et al. [36]	The majority (86%) of neonates required high-frequency ventilation, and half (50%) needed ECMO for a median of 7 days. All neonates required patch repair for their diaphragmatic hernia. Other morbidities included a 5-minute Apgar score less than 7 in 50% of neonates, 36% experienced postoperative complications (re-herniation due to patch dehiscence), and 50% required supplemental oxygen or treatment for PH at discharge. 2 infants required tracheostomy	N/A
2020, Wada et al. [37]	3 cases (27%) of PPROM. 2 cases required EXIT due to difficulties in balloon removal. In 1 case, balloon puncture was unsuccessful, necessitating elective EXIT	N/A
2020, Cruz-Martínez et al. [38]	Preterm delivery (PTD <37 weeks) occurred in 68% of the FETO group, and a longer length of stay in the NICU (25.4 ± 18.5 days) compared to the control group. PPROM occurred in 56% of cases. All neonates required intubation, and some needed HFOV and iNO	The study reports no survivors in the control group
2022, Cruz-Martínez et al. [43]	The FETO group had significantly shorter durations: ventilatory support (17.8 days vs. 32.3 days in controls), NICU stay (34.2 days vs. 58.3 days in controls). However, they also experienced a significantly higher incidence of PPROM (37.9% vs. 13.8% in controls) and preterm delivery (72.4% vs. 37.9% in controls)	The control group had longer durations of ventilatory support and NICU stay compared to the FETO group. A lower incidence of PPROM and preterm delivery was observed
2021, Russo et al. [44]	In the FETO group with severe pulmonary hypoplasia, there was no significant difference in neonatal morbidity compared to the expectantly managed group. However, there was a higher incidence of PPROM and lower gestational age at birth. 2 infants had tracheomalacia	In the expectantly managed group with severe pulmonary hypoplasia, survivors had a significantly longer NICU stay compared to those with mild hypoplasia. There was no significant difference in other morbidity indicators (need for patch surgery, treatment for PH, PH on day 28, need for ECMO)
2022, Donepudi et al. [42]	87.5% (7/8) resolution of PH by hospital discharge; 50% (4/8) needed ECMO. A higher proportion of infants in this group had evidence of PH on the first postnatal echocardiogram (88.9%). 88.9% required iNO	40% (8/20) resolution of PH by hospital discharge; 71.4% (15/21) needed ECMO. A higher proportion of infants in this group had evidence of PH on the first postnatal echocardiogram (95.2%). 76.2% required iNO
2021, Deprest et al. [39]	The FETO group showed a higher incidence: preterm, prelabor rupture of membranes (44%), preterm birth (64%), bronchopulmonary dysplasia (65% of survivors to NICU discharge), PH (74% of survivors to NICU discharge), neonatal death (1/91) due to balloon removal complications, tracheomalacia (2/91)	The expectant care group showed a lower incidence of the morbidities: preterm, prelabor rupture of membranes (12%), preterm birth (22%), bronchopulmonary dysplasia (65% of survivors to NICU discharge), PH (67% of survivors to NICU discharge), neonatal death (38/95)
2021, Deprest et al. [40]	The FETO group experienced a higher incidence of tracheomalacia (2/40), and higher rates of bronchopulmonary dysplasia (75%), PH (94%), and sepsis (62%) among survivors	Among survivors, there were higher rates of PH (100%) and sepsis (100%)
2022, Van Calster et al. [45]	Increased prematurity (earlier gestational age at delivery). Early balloon insertion, in particular, strongly increased the risk of preterm delivery	N/A
2023, Sferra et al. [46]	Significantly lower median O/E LHR prenatally; 58% remained on bronchodilators/inhaled corticosteroids at follow-up; 67% were feeding tube dependent at follow-up; increased rates of gastrostomy/jejunostomy at discharge and follow-up; high rates of GERD requiring fundoplication in some cases; longer hospital stays	Higher median O/E LHR prenatally; 67% remained on bronchodilators/inhaled corticosteroids at follow-up; 63% were feeding tube dependent at follow-up; higher ECMO utilization rate; shorter hospital stays
2024, Wild et al. [47]	8/12 (66.7%) infants were born prematurely (<35 weeks gestational age), and 8/12 (66.7%) experienced PPROM. ECMO use was 25%. There were 5 infants who required emergent balloon removal (2 EXIT and 3 tracheoscopic removals). Tracheomegaly was a known side effect. The median duration of delivery room stabilization was 16 minutes longer than in the SOC group	3/35 (8.6%) infants were born prematurely (<35 weeks gestational age), and 3/35 (8.6%) experienced PPROM. ECMO use was 60%
2024, Bergh et al. [48]	Higher rates of PPROM (54.0% vs 14.3% in the control group), earlier gestational age at delivery (median 35.0 weeks vs. 38.3 weeks in the control group), and Lower birth weight (mean 2,487 g vs. 2,857 g in the control group). In a sub-analysis of the most severe cases, FETO patients required fewer days of ECMO (median 9.0 days vs. 17.0 days in the control group)	Lower rates of PPROM (14.3%), later gestational age at delivery (median 38.3 weeks), higher birth weight (mean 2,857 g), and Longer ECMO duration (median 17 days in the most severe cases)
2024, Dütemeyer et al. [49]	The FETO group showed a higher rate of preterm birth (72.34%), less frequent use of ECMO (4.26%), a trend toward more infants having PPHN at discharge and needing feeding support at 1 year of life, although not statistically significant	The expectant management group showed a lower rate of preterm birth (29.93%), more frequent use of ECMO (55.78%), more frequent corrective surgery (85.03%), and a lower weight Z-score at 1 year of life
2024, Sevilmis et al. [50]	Surfactant recipients had significantly worse prenatal prognostic features (O/E TFLV, O/E LHR, percent liver herniation), lower Apgar scores, increased ECMO use, increased length of stay, and increased rates of HFOV. In the non-FETO group, surfactant recipients had higher rates of ECMO and HFOV	In the non-FETO group, the control group (those who did not receive surfactant) had lower rates of ECMO and HFOV use compared to the surfactant group. The control group also had shorter hospital stays
2024, Sferra et al. [51]	Among the FETO group, there was notable concern for language delay at 12 months (CLAMS median DQ 80.1), although this improved by 24 months. At 12 months, there was also concern for visual-motor and problem-solving delays (CAT median DQ 81.3), which also improved by 24 months. A high percentage (56%) required ECLS. A majority continued to face gastroesophageal reflux and other oral feeding challenges	The control group (high-severity non-FETO) exhibited worsening neurodevelopmental delays (NDD) from 12 to 24 months, with 60% showing language delay and 40% displaying motor delay at 24 months. A high percentage (89%) required ECLS
2024, Manfroi et al. [52]	The most frequent complication was premature delivery (56.5% of cases). PPROM in <34 weeks occurred in 26% of cases, and 50% had PPROM in <37 weeks. PH was present in 80.6% of live births. The main causes of neonatal death were respiratory failure and cardiac dysfunction (85% occurring within the first 20 days)	N/A
2024, Olutoye et al. [53]	Neonatal morbid outcomes included: Tracheomegaly (in 24/34 patients), The need for reintubation to upsize the endotracheal tube (in 8/34 patients), Additional ENT or pulmonology procedures (in 9/34 patients, including airway endoscopies), Tracheostomy (in 8/34 patients), ECMO use (in 14/34 patients), and The need for sildenafil and supplemental oxygen at discharge (in 13/34 patients each). Tracheomalacia was also observed in 9/34 patients	N/A

**Table 4:** Maternal outcomes among the intervention and/or control groups iPPROM: iatrogenic preterm premature rupture of membrane, PROM: premature rupture of membrane, PPROM: preterm premature rupture of membrane, FETO: fetoscopic endoluminal tracheal occlusion, N/A: not applicable, GTN: glyceryl trinitrate

Year, author	Maternal outcome/s among the intervention group	Maternal outcome/s among the control group
2003, Harrison et al. [9]	All women require tocolysis for uterine contractions. 7 of 11 (64%) experienced ultrasonographically detectable chorioamniotic separation. All eleven women experienced PPROM. The mean gestational age at delivery was significantly lower (30.8 ± 2.0 weeks) compared to the control group. 1 woman had a maternal wound infection. 3 women had mild pulmonary edema requiring supplemental oxygen for less than 48 hours	3 of 13 (23%) experienced PPROM. 4 of 13 (31%) experienced preterm labor. 1 woman experienced placental abruption
2006, Jani et al. [13]	There were no serious maternal complications such as hemorrhage, pulmonary edema, or infection. However, 14 (58.3%) experienced iPPROM after 37 weeks	The only maternal outcome reported for the control group is the antenatal loss rate of 13% (5 of 37), which includes terminations of pregnancy
2011, Deprest et al. [12]	There were no maternal complications such as hemorrhage, placental abruption, pulmonary edema, or infection in the FETO group. However, 11 (52.4%) experienced postoperative PPROM	Maternal outcomes are not explicitly reported for the control groups
2007, Saura et al. [19]	41.6% incidence of PPROM in the FETO group	N/A
2006, Jani et al. [20]	Procedure-related maternal complications occurred in 7 cases, including 1 intra-amniotic hemorrhage requiring a blood transfusion and 5 cases of chorioamnionitis following PPROM	N/A
2011, Peralta et al. [21]	There were no maternal or fetal complications during FETO or balloon removal in the 8 cases analyzed. PPROM occurred in 5 of the 8 cases (62.5%), typically after the procedure for TO reversal. Preterm labor occurred in 3 of these 5 cases	N/A
2012, Ruano et al. [14]	No maternal deaths, blood transfusions, or infections occurred in the FETO group. PPROM occurred in 6 (35.3%) cases, extremely preterm delivery in 3 (17.7%), and placental abruption in 1 (5.9%)	No maternal deaths, blood transfusions, or infections occurred in the control group. PPROM occurred in 5 (27.8%) cases, and extremely preterm delivery in 2 (11.1%)
2012, Ruano et al. [22]	1 case of maternal infection (chorioamnionitis after PPROM) occurred. There were also instances of PPROM and preterm delivery, but these did not differ significantly from the control group	No maternal deaths, blood transfusions, or abruptions occurred. There were instances of PPROM and preterm delivery; however, these were not significantly different from those in the intervention group
2013, Ruano et al. [23]	Maternal and fetal demographic characteristics and obstetric complications (including maternal infection, placental abruption, and PPROM) were similar between the early FETO and standard FETO groups. No maternal blood transfusions were required in either group	Similar to the intervention groups, no maternal blood transfusions were required. There were no cases of maternal infection or placental abruption. PPROM occurred in 20% of cases
2013, Ali et al. [24]	The primary maternal outcome reported is the high rate of premature delivery (84%). The study also notes that women who went into premature labor were given oral nifedipine or GTN patches	N/A
2014, Engels et al. [26]	PPROM rate (48%) and gestational age at delivery (median 36.1 weeks)	PPROM rate (39%) and gestational age at delivery (median 35.4 weeks)
2017, Braga et al. [28]	The study reports no hemodynamic or respiratory changes, nor any maternal complications related to anesthesia. The most common complications related to the procedure itself were loss of amniotic fluid into the maternal peritoneal cavity (1 case) and PROM (3 cases)	N/A
2017, Belfort et al. [32]	No maternal complications or fetal deaths related to the procedure were reported. Preterm prelabor rupture of membranes occurred in 3 of 11 (27.3%) patients	No maternal outcomes are explicitly reported for the historical control group
2020, Baschat et al. [36]	There were no procedure-related maternal complications such as uterine wall bleeding or chorioamnionitis. However, obstetric interventions were frequently required to manage preterm birth risk. These included additional tocolytics in 79% of cases, cervical pessary placement in 21%, and amnioreduction in 36%. PPROM occurred in 29% of cases, and preterm labor without PROM in 36%. The median gestational age at birth was 39 2/7 weeks	N/A
2020, Cruz-Martínez et al. [38]	No maternal or fetal complications were observed during the FETO procedure. PPROM occurred in 56% of cases. Median maternal discharge time was 1 day.	N/A
2022, Cruz-Martínez et al. [43]	Maternal outcomes in the FETO group included a higher incidence of PPROM (37.9%), a higher incidence of preterm delivery (72.4%), a lower median gestational age at delivery (35.2 weeks), and a median maternal discharge time of 1.9 days	Maternal outcomes in the control group included a lower incidence of PPROM (13.8%), a lower incidence of preterm delivery (37.9%), and a higher median gestational age at delivery (37.1 weeks)
2021, Russo et al. [44]	In the FETO group, there was a higher incidence of PPROM. 1 case developed complete separation of the chorionic membranes	Antepartum bleeding and maternal infection occur in a small number of cases
2022, Donepudi et al. [42]	No significant differences were found in maternal age or parity compared to the control group. However, women in the FETO group had a shorter median gestational age at delivery (35 weeks) and lower birth weights (2101 g) than the non-FETO group	No significant differences were found in maternal age or parity between the intervention group and the control group. However, women in the non-FETO group had a longer median gestational age at delivery (38 weeks) and higher birth weights (3050 g) than the FETO group
2022, Trad et al. [41]	The primary maternal outcomes reported are gestational age at delivery, PPROM, and the interval between intervention and delivery. There were no statistically significant differences in maternal age, race, gravidity, or parity between the 3 intervention groups	N/A
2021, Deprest et al. [39]	The FETO group experienced a significantly higher rate of preterm, prelabor rupture of membranes, and preterm birth. There were no cases of placental abruption in this group	The expectant care group had a significantly lower rate of preterm, prelabor rupture of membranes, and preterm birth compared to the FETO group. There were no cases of placental abruption
2021, Deprest et al. [40]	The incidence of preterm, prelabor rupture of membranes was significantly higher in the FETO group (47%) compared to the expectant care group (11%). The incidence of preterm birth was also significantly higher in the FETO group (75%) compared to the expectant care group (29%). There was 1 case of placental abruption and 1 case of placental laceration (resulting in a neonatal death)	The incidence of preterm, prelabor rupture of membranes was 11%, and the incidence of preterm birth was 29%. There was 1 case of placental abruption

Discussion

Summary of Main Results

We included 40 studies assessing FETO as a prenatal intervention for CDH, which were the focus of this systematic review. The findings clarify both the potential benefits and the risks associated with FETO compared to expectant management. Although survival outcomes improved in many trials, particularly for severe cases of CDH, infant morbidity and maternal challenges remain significant concerns. The results suggest that in severe cases of CDH, FETO may improve survival rates; however, its effectiveness depends on the gestational age at the time of intervention, disease severity, and postnatal care practices. Additionally, maternal and neonatal complications, especially preterm birth and pulmonary hypertension, continue to pose major challenges.

Impact of Study Designs on Outcomes

RCTs (strongest evidence for FETO's efficacy): RCTs provide the strongest evidence of FETO's safety and efficacy compared to expectant management. RCTs that assigned individuals to either FETO or conventional postnatal care were conducted by Harrison et al. [9], Cortes et al. [18], Ruano et al. [22], and Deprest et al. [39]. Key findings from these trials include higher survival rates in FETO-treated newborns, particularly in severe cases with liver herniation and low LHR. However, variability in postnatal outcomes was noted; some trials, especially those involving mild cases of CDH, showed no significant survival benefit. An increased risk of preterm birth and PPROM was also reported, contributing to neonatal complications. Although these RCTs demonstrate the potential of FETO, the variability in outcomes underscores the importance of careful patient selection.

Prospective and retrospective cohort studies (actual FETO data): Cohort studies have provided valuable insights into the long-term outcomes and complications associated with FETO. Prospective cohort studies by Jani et al. [13], Jani et al. [20], and Doné et al. [25] confirmed that FETO improves neonatal lung function and increases survival, particularly in severe CDH cases. Retrospective studies by Ali et al. [29], Kosinski et al. [30], and Jiménez et al. [31] reported significantly higher rates of preterm birth in FETO groups, raising concerns about long-term morbidity. Standardizing surgical techniques may enhance consistency in outcomes, as demonstrated by multicenter studies by Engels et al. [26], Russo et al. [44], and Bergh et al. [48], which have shown variability in FETO results depending on center-specific experience and protocols.

Single-center vs. multicenter studies (influence of institutional expertise): Single-center trials, such as those by Belfort et al. [32] and Sevilmis et al. [50], have demonstrated high survival rates but also high rates of preterm birth and PPROM, suggesting that institutional expertise plays a crucial role in FETO success. Multicenter studies by Van Calster et al. [45] and Deprest et al. [39] revealed significant differences in survival rates depending on postnatal care practices, highlighting the need for international guidelines to optimize outcomes.

Effect of Intervention Timing on Neonatal Outcomes

Examining FETO timing techniques and outcomes, two main FETO timing techniques were examined in the reviewed papers: early FETO (22-24 weeks of gestation) and standard FETO (26-30 weeks of gestation). Notably, although tracheal complications were more common, Ruano et al. [23] reported slightly higher survival rates in early FETO cases (62.5%) compared to standard FETO. However, Van Calster et al. [45] observed an increased likelihood of very preterm birth (<32 weeks) in the early FETO group, raising concerns about neonatal respiratory distress and long-term pulmonary function. According to Deprest et al. [39], standard FETO improved survival rates without significantly increasing the risk of PPROM, suggesting that later gestational intervention may better balance risks and benefits. These findings suggest that FETO scheduling should be individualized, taking into account fetal lung maturity, defect severity, and maternal health factors.

Comparative outcomes (FETO vs. expectant management): Most studies demonstrated improved survival with FETO, particularly in severe CDH cases characterized by a LHR <1.0 and liver herniation. Ruano et al. [22] and Deprest et al. [39] showed a two- to threefold increase in survival compared to expectant management. However, Dütemeyer et al. [49] found higher survival rates in the expectant management group (74.3% vs. 44.7%), suggesting that certain patients might benefit more from postnatal intensive care than from prenatal intervention.

Pulmonary hypertension remains a significant concern, with Jiménez et al. [31] and Doné et al. [25] reporting incidence rates above 50% in FETO-treated neonates. Sferra et al. [46] also noted frequent gastrointestinal and tracheal complications, with 67% of FETO newborns requiring feeding tube support during follow-up. Preterm birth rates were markedly higher in FETO groups, especially when the procedure was performed before 26 weeks, as reported by Van Calster et al. [45].

PPROM rates ranged from 30% to 75% among FETO-treated pregnancies. Deprest et al. [39] reported a nearly fourfold increase in PPROM risk compared to expectant management. While Ruano et al. [14] found no major maternal complications such as infection or hemorrhage, the elevated rate of preterm labor raises concerns about risks in future pregnancies.

Neonatal survival outcomes (FETO vs. expectant management): This systematic review examined neonatal survival outcomes in fetuses undergoing FETO vs. expectant management. The findings reveal substantial variations in survival rates, influenced by study design, gestational age at intervention, postnatal care strategies, and disease severity.

Overall Survival Trends: Does FETO Improve Neonatal Survival?

RCTs and prospective studies: Ruano et al. [22] reported a 50% survival rate in the FETO group compared to only 4.8% in the expectant management group, indicating a tenfold improvement in survival. Deprest et al. [39] found that 40% of infants survived to discharge after FETO, while only 15% stayed in the expectant group, reinforcing FETO's potential role in improving survival in severe CDH cases. Belfort et al. [32] demonstrated a 70% one-year survival rate in FETO-treated infants vs. 11% in the control group, highlighting a significant advantage in appropriately selected cases.

Retrospective and observational studies: Saura et al. [19] reported a 53.8% survival rate in FETO-treated infants but a higher survival rate of 83.3% in the non-FETO group, suggesting that some patients may benefit more from postnatal management than from fetal intervention. Dütemeyer et al. [49] found that only 44.7% of FETO infants survived to discharge, compared to 74.3% in the expectant management group, raising concerns about the complication rates associated with the procedure.

Gestational Age and Timing of Intervention

Ruano et al. [23] reported a 62.5% survival rate with early FETO compared to 0% with expectant management, supporting earlier intervention in severe cases. However, Van Calster et al. [45] found that early FETO increased the risk of preterm birth, thereby compromising survival outcomes. Standard FETO, performed at 27-29 weeks, showed a 63% survival rate according to Deprest et al. [39], suggesting that later intervention may better balance lung development with the risk of extreme prematurity. These findings indicate that the optimal timing of FETO remains uncertain, highlighting the importance of personalized decision-making.

Disease Severity and Survival

In severe CDH cases, particularly in fetuses with a low LHR and liver herniation, FETO is most effective. Jani et al. [13] observed a 50% survival rate in FETO-treated severe CDH cases, compared to only a 9% survival rate in non-FETO cases. Supporting its efficacy in cases of severe lung hypoplasia, Cruz-Martínez et al. [38] reported a 41% survival rate in fetuses with an observed/expected LHR of 45% who underwent FETO vs. 15% in those who did not. These findings support prioritizing FETO for the most severe cases of CDH while carefully evaluating its benefits in moderate cases.

Neonatal Morbidity Outcomes: FETO vs. Expectant Management

With major concerns about pulmonary hypertension, preterm birth, feeding problems, and long-term neurodevelopmental deficits, morbidity outcomes among neonates undergoing FETO have varied substantially between studies. Compared to expectant management, FETO may improve survival but also increases the risk of respiratory and gastrointestinal complications, which require thorough postnatal management.

Pulmonary and Respiratory Morbidity: The Leading Concern

Ruano et al. [14] found that 47.1% of FETO-treated infants suffered from severe pulmonary hypertension, compared to 88.9% in the expectant management group, indicating that FETO may lower the incidence but does not eliminate the risk. Similarly, Ruano et al. [22] reported that 50% of FETO newborns had severe pulmonary arterial hypertension, compared to 85.7% under expectant care, suggesting a potential benefit of FETO while still highlighting significant morbidity. Although survival rates have improved, Deprest et al. [39] observed that 74% of FETO survivors had pulmonary hypertension, compared to 67% in the expectant management group, indicating no substantial improvement in this outcome.

Need for Respiratory Support and ECMO

Belfort et al. [32] determined that 30% of infants treated with FETO required ECMO, whereas 78% of the control group did, suggesting that FETO may reduce ECMO dependency in some cases. Conversely, Dütemeyer et al. [49] reported that ECMO utilization was lower in the expectant management cohort (4.26%) compared to the FETO cohort (55.78%), indicating that some neonates treated with FETO still require intensive postnatal support. Cruz-Martínez et al. [38] found that all FETO infants required intubation and high-frequency ventilation, highlighting the considerable respiratory challenges despite in-utero intervention. These findings suggest that while FETO may promote lung development, it does not fully prevent respiratory complications, underscoring the need for rigorous neonatal respiratory management.

Prematurity and Associated Complications

Deprest et al. [39] reported PPROM in 44% of FETO cases, compared to 12% under expectant management, thereby increasing the risk of preterm birth and neonatal complications. Van Calster et al. [45] found that earlier balloon insertion further elevated the risk of preterm delivery, highlighting the critical importance of FETO timing to avoid extreme prematurity. Supporting the association between FETO and premature birth, Bergh et al. [48] reported a median gestational age at birth of 35 weeks in the FETO group vs. 38.3 weeks in the expectant management group. Despite the heightened risk of prematurity, Cruz-Martínez et al. [43] observed that FETO neonates had shorter NICU stays (34.2 vs. 58.3 days) and required less ventilatory support (17.8 vs. 32.3 days) compared to those managed expectantly, suggesting potential postnatal benefits. However, concerns remain regarding long-term respiratory outcomes; Donepudi et al. [42] reported higher rates of bronchopulmonary dysplasia (65%) and tracheomalacia (two out of 40 cases) in FETO newborns. These findings indicate that while FETO may promote lung development, the associated risks of prematurity necessitate careful maternal monitoring and neonatal stabilization.

Gastrointestinal and Feeding Challenges

Many FETO-treated babies experienced gastrointestinal problems and feeding difficulties, often requiring ongoing nutritional support. With higher rates of gastrostomy and jejunostomy, Sferra et al. [46] found that 67% of FETO-treated newborns remained dependent on feeding tubes. Emphasizing the importance of early postnatal gastrointestinal evaluation, Ruano et al. [22] reported a case in which a FETO newborn died from aspiration pneumonia due to severe esophageal reflux. Highlighting the high incidence of GERD requiring fundoplication, Belfort et al. [32] noted that 50% of FETO survivors needed supplemental oxygen at six months. These findings suggest that both prematurity and the lung expansion mechanisms associated with FETO may contribute to gastrointestinal complications, necessitating comprehensive postnatal feeding therapy.

Neurodevelopmental Outcomes and Long-Term Follow-Up

Long-term neurodevelopmental problems have been observed in FETO-treated newborns. Although some improvements were noted by 24 months, Sferra et al. [51] reported linguistic deficits in 56% of FETO infants at 12 months. In a study of 28 FETO survivors, Ali et al. [35] identified delayed motor milestones in one case; however, no significant difference was found in overall neurological outcomes compared to expectant care. Harrison et al. [9] found white matter damage in five of eight FETO survivors, similar to findings in control infants, suggesting that preterm birth and the severity of CDH, rather than the FETO procedure itself, may be the primary contributors to neurodevelopmental abnormalities. These findings underscore the importance of long-term neurodevelopmental follow-up in FETO-treated newborns, particularly concerning motor performance, cognitive function, and speech development.

Maternal Outcomes: Risks Associated With FETO vs. Expectant Management

Long-term neurodevelopmental problems have emerged in newborns who underwent FETO. Concerning PPROM, preterm labor, and procedural risks, maternal outcomes associated with FETO raise significant concerns. Although FETO aims to improve neonatal survival in cases of severe CDH, its impact on maternal health and pregnancy complications must be carefully evaluated.

PPROM and Preterm Birth

Long-term neurodevelopmental problems have emerged in newborns who underwent FETO. PPROM is the most common maternal complication following FETO, often leading to preterm labor and delivery. Deprest et al. [39] reported that FETO accounted for 47% of PPROM cases, compared to 11% in expectant management, suggesting that membrane instability is associated with the procedure. Similarly, Ruano et al. [14] found that 35.3% of women treated with FETO experienced PPROM, compared to 27.8% in the expectant care group. While both groups are at risk, FETO appears to increase the likelihood of PPROM. Cruz-Martínez et al. [38] observed that 56% of FETO pregnancies, with a median gestational age of 35 weeks, experienced PPROM, often resulting in moderate-to-late preterm births. The fetoscopic intervention, which involves invasive transuterine procedures that may disrupt the fetal membranes, likely contributes to the higher incidence of PPROM in FETO pregnancies. Further research is needed on tocolytic therapy and membrane-sealing techniques, such as collagen plug implantation.

Gestational Age at Delivery: FETO vs. Expectant Care

Long-term neurodevelopmental problems have emerged in newborns who underwent FETO. Deprest et al. [39] reported that the mean gestational age at birth in FETO pregnancies was 30.8 weeks, compared to 37.1 weeks in the expectant group, highlighting the risk of neonatal complications associated with prematurity. Under expectant care, the median gestational age at delivery in FETO pregnancies was 38.3 weeks; in the study by Bergh et al. [48], it was 35.0 weeks. Preterm birth limits fetal development, as evidenced by the significantly lower median birth weight in FETO pregnancies (2101 g) compared to expectant management (3050 g). Preterm birth is a substantial risk with FETO; however, some studies suggest that tocolysis and perinatal care may help prolong pregnancy. To reduce the risk of preterm birth, 79% of FETO pregnancies reported by Baschat et al. [36] received tocolytics.

Procedure-Related Maternal Risks: Infection, Bleeding, and Anesthesia Complications

Although most studies did not report significant maternal complications related to FETO surgery, a few minor concerns were noted. Jani et al. [20] recorded seven cases of maternal complications, including five episodes of chorioamnionitis following PPROM and one case of intra-amniotic hemorrhage requiring transfusion. Ruano et al. [22,23] reported no instances of maternal hemorrhage, sepsis, or transfusion, suggesting that the risk of infection is primarily secondary to preterm rupture of membranes, even though chorioamnionitis was observed in some patients following PPROM. Baschat et al. [36] reported no cases of uterine wall hemorrhage or chorioamnionitis, emphasizing that with proper procedural technique, major maternal risks are rare. While serious maternal complications are uncommon, the risks of infection and bleeding should not be overlooked, particularly in settings with limited fetoscopic expertise.

Strengths and limitations

Strengths of this review include the use of a comprehensive search strategy across multiple databases and adherence to the PRISMA guidelines. The inclusion of a wide range of study designs provided a broad overview of FETO outcomes in CDH. However, limitations include heterogeneity among the included studies in terms of patient selection, timing of intervention, and outcome measures, which restricted the ability to perform a meta-analysis. Several studies were retrospective and had small sample sizes. Additionally, publication bias and language restrictions may have influenced the results. Further high-quality randomized controlled trials are needed.

## Conclusions

Though treatment improves survival rates in severe cases of CDH, the results of this systematic study indicate that FETO is associated with increased neonatal morbidity and maternal complications. These findings highlight the importance of strict patient selection criteria to maximize benefits while minimizing risks. In clinical practice, FETO should be reserved for the most severe cases of CDH, where the potential survival benefits outweigh the possible drawbacks. Future research should focus on standardizing FETO procedures, improving postnatal care systems, and exploring alternative therapies that reduce the risks of preterm birth and associated morbidity.
